# Immediate and Delayed Effects of Forearm Kinesio Taping on Grip Strength

**DOI:** 10.5812/ircmj.19797

**Published:** 2014-08-05

**Authors:** Hosein Kouhzad Mohammadi, Khosro Khademi Kalantari, Sedighe Sadat Naeimi, Mohammad Pouretezad, Esmaeil Shokri, Mojdeh Tafazoli, Mahboobeh Dastjerdi, Leila Kardooni

**Affiliations:** 1 Department of Physical Therapy, Faculty of Rehabilitation Sciences, Shahid Beheshti University of Medical Sciences, Tehran, IR Iran; 2 Department of Physical Therapy, Faculty of Rehabilitation Sciences, Ahvaz Jundishapur University of Medical Sciences, Ahvaz, IR Iran

**Keywords:** Athletic Tape, Hand Strength, Forearm

## Abstract

**Background:**

Due to the fundamental role of gripping in most upper limb activities, grip strength promotion is a chief goal in the treatment of patients with upper limb musculoskeletal disorders. Kinesio taping is a novel and effective therapeutic technique believed to facilitate muscle contraction through stimulating mechanoreceptors and increasing the sensory feedback around the taped region.

**Objectives:**

The present study aimed to identify the best region (flexor, extensor and flexor/extensor regions) and time (immediate, 0.5, 1, 1.5, and 2 hours) of forearm Kinesio taping to obtain the maximum improvement in grip strength.

**Materials and Methods:**

In this longitudinal study, 40 healthy men and women (the mean age of 22.3 ± 2.19 years) were selected among students of Shahid Beheshti University of Medical Sciences, Tehran, Iran by simple, nonrandom sampling method. A dynamometer was used to measure grip strength immediately and every 30 minutes during the two hours after I-shaped application of tape (with 50% stretch) to the flexor, extensor, and flexor/extensor forearm muscles.

**Results:**

Grip strength was significantly increased in various muscle groups for males (P = 0.002) and females (P = 0.000) of the forearm and at different intervals for males (P = 0.000) and females (P = 0.000). Moreover, in both men and women, tape application to the extensor region provided greater grip strength compared to taping of the flexor and flexor/extensor regions (P = 0.000 for both). Furthermore, the maximum increase in grip strength were 0.5 (10.8% increase, P = 0.001) and 1.5 h (23.9% increase, P = 0.000) after taping in males and females, respectively.

**Conclusions:**

Taping the extensor region of forearm is recommended to achieve higher grip strength. Although grip strength increased at a slower pace in females than males, the final values were higher in women.

## 1. Background

Grip strength measures the ability of hand muscles to grip, and is frequently used to assess upper-limb impairment or to develop a suitable treatment plan (1-3). Grip strength is influenced by age, sex, body mass index, occupation, leisure, physical activities, upper limb muscle strength, diet, pain levels, and loss of sensory function (1, 2, 4-6).

Previous studies found that if neuromuscular control and accuracy of proprioception are affected by muscle fatigue, or muscle loses its strength (7, 8), forearm muscle is put at risk of injury (9).

Kinesio tape is made of elastic adhesive materials frequently used in the prevention of sport injuries, rehabilitation of injured athletes, and enhancing athletes’ performance (10, 11).

Kinesio tape improves proprioception by providing constant cutaneous afferent stimulation of the skin. This activity improves joint function, stimulates sensory mechanisms, decreases pain as a result of a reduction in neurological activation, enhances blood and lymph circulation to the local area by lifting fascia and soft tissue, realigns fascia tissue function through normalizing muscle tension, and improves muscle function (12-14).

However, the effect of Kinesio taping on muscle strength remains a controversial issue. While some researchers revealed beneficial effects of Kinesio taping on muscle strength (15-17), others considered it as an ineffectual measure (18-20). There is little evidence about the effect of Kinesio taping on grip strength. However, the effect of Kinesio taping on grip strength remains a controversial issue.

Several studies investigated immediate effect of Kinesio taping on the maximum grip strength (21-24), but none of them compared the effect of Kinesio taping on three regions of forearm (flexor, extensor and flexor/extensor together). Chang et al. (21) and Lee et al. (23) assessed the effect of taping in flexor region, Kuo et al. (22) in extensor region and Donec et al. (24) in flexor/extensor regions.

Some studies evaluated immediate effects of taping (21, 23); only one study assessed its effect immediately and after 24 hours of taping (22) and one study reported the effect of taping after 30 and 60 minutes (24).

Therefore, none of these studies investigated delayed effects of taping in a consecutive period to determine the peak time of taping effect. Therefore, there is a lack of study investigating the best region and time interval to obtain the maximum grip strength.

## 2. Objectives

The current study aimed to investigate immediate and delayed (0.5, 1, 1.5, and 2 hours) effects of Kinesio taping to flexor, extensor, and flexor/extensor regions of the forearm.

## 3. Materials and Methods

### 3.1. Participants

This was a longitudinal study. Forty healthy volunteers (20 males and 20 females) from 52 students were selected according to the inclusion and exclusion criteria. Inclusion criteria included non-athletes, age between 20-30 years and BMI between 22-25 kg/m^2^. Exclusion criteria included any allergy to Kinesio tape, limited range of motion, deformities, fractures, arthritis, tendonitis of the upper extremity joints and any systemic or neurological disorders affecting the grip strength within the last six months.

The study protocol was approved by the Ethics Committee of Shahid Beheshti University of Medical Sciences (Tehran, Iran). The code of ethics approval was 1391-1-93-10450 and the date was 2013/10/6.

All subjects were given written information about the aims of the study and if willing were asked to sign a consent form. Subjects were informed that there is no harm in this study and they could leave the study at any time.

### 3.2. Sampling Method

Participants were selected from Shahid Beheshti University of Medical Sciences through simple, nonrandom sampling method.

### 3.3. Data Collection

For better adherence of the Kinesio tape, all participants were asked to shave their dominant forearm, wash it with soap, and dry it. The flexor, extensor, and flexor/extensor regions of the forearm were separately taped with 48-hour intervals. Participants sat on a straight back chair, and were asked to assume an upright sitting position in which both feet were on the floor and the dominant forearm was held against the body with the elbow flexed at 90° and the forearm and wrist in the neutral position. They were instructed to grip the handle of the dynamometer (Digital Pinch/Grip Analyzer, MIE, England) as tightly as possible for three seconds and the values were recorded. Force (in kg) was the main variable measured in this study. The equipment used was calibrated before conducting the study by the provider company technician. After each experiment, the equipment was reset by zero button. Measurements were performed in triplicate with sufficient intervals. For each participant, the three values were averaged and regarded as the maximum grip strength before taping. The same procedure was repeated immediately and 0.5, 1, 1.5, and 2 hours after I-shaped application of Kinesio taping (TEMTEX, South Korea, 5 cm wide).

To ensure uniform stretching (50%) among all subjects, for flexor muscles the distance between the medial epicondyle of the elbow and the anterior line of the wrist and for extensor muscles the distance between the lateral epicondyle of the elbow and the styloid process of the radius were measured and multiplied by 0.83. Obtained values were considered as the length of the required Kinesio tape. All measurements were performed by a qualified physiotherapist with at least 8-year experience of taping.

### 3.4. Data Analysis

Data was analyzed using SPSS for Windows 16.0 (SPSS Inc, Chicago, IL, USA). First, Kolmogorov-Smirnov test was used to check the normal distribution of data. Since the results confirmed normal distribution of all variables, parametric tests were applied for statistical analyses. Therefore, repeated measures analysis of variance (ANOVA) was conducted to compare grip strength at different intervals (immediately, 0.5, 1.0, 1.5, and 2 hours after taping), and various muscle groups of the forearm (flexor, extensor, and flexor/extensor regions). Moreover, Bonferroni correction was applied for multiple comparisons between different intervals and various muscle groups of the forearm. P-values less than 0.05 were considered significant.

## 4. Results

Forty right-handed subjects (20 males and 20 females) with the mean age of 22.30 ± 2.19 years, the mean weight of 66.03 ± 12.76 kg, and the mean height of 167.53 ± 8.80 cm participated in the present study. No significant differences were found in age, weight and height between the two genders (P > 0.05 in all instances). Sample characteristics were provided in Table 1.

**Table 1.** Sample Characteristics ^a,b^

	Age, y	Weight, kg	Height, cm	BMI, kg/m^2^
**Male (n = 20)**	22.35 (1.95)	74.45 (10.96)	173.70 (7.33)	23.57 (0.97)
**Female (n = 20)**	22.25 (2.46)	57.60 (8.06)	161.35 (5.00)	23.06 (0.88)

^a^ Values are presented as mean (SD).

^b^ Abbreviation: BMI; body mass index.

In both genders, grip strength was significantly increased at different times and regions (P < 0.05 in all instances). Detailed information, including changes in different intervals and regions in both genders with their P values were demonstrated in Table 2.

**Table 2.** Percent Changes (Compared to Before Taping) in Grip Strength After Application of Kinesio Tape on Flexor, Extensor and Flexor/Extensor Regions at Different Times in Males and Females ^a^

Time interval	Male			Female		
	Flexor	Extensor	Flexor/Extensor	Flexor	Extensor	Flexor/Extensor
**Immediate**	1.67 (1.83), P < 0.001	5.07 (5.52), P < 0.001	1.92 (1.59), P < 0.001	0.31 (5.14), P = 0.009	7.59 (6.20), P < 0.001	0.81 (3.80), P = 0.050
**0.5 h**	4.13 (2.31), P < 0.001	10.83 (7.88), P < 0.001	4.17 (3.65), P < 0.001	1.76 (2.40), P < 0.001	13.53 (8.76), P < 0.001	1.58 (2.78), P = 0.019
**1 h**	3.52 (3.32), P < 0.001	9.21 (8.33), P < 0.001	3.13 (4.20), P = 0.002	4.43 (3.02), P < 0.001	18.50 (13.92), P < 0.001	3.98 (3.13), P < 0.001
**1.5 h**	2.29 (3.69), P = 0.041	5.42 (6.40), P = 0.015	2.92 (3.46), P = 0.049	7.79 (3.20), P < 0.001	23.92 (15.96), P < 0.001	7.87 (3.11), P < 0.001
**2 h**	1.83 (3.55), P = 0.029	1.68 (2.99), P = 0.038	2.63 (2.29), P < 0.001	4.91 (2.88), P < 0.001	18.86 (15.02), P < 0.001	4.70 (2.70), P < 0.001

^a^ Values are presented as mean (SD).

Repeated measurements analysis demonstrated a significant difference between different times after taping in males (P = 0.000). Further analysis using Bonferroni correction for Post Hoc tests indicated no significant differences between immediate and 1 hour (P = 0.87), immediate and 1.5 hours (P = 0.13) in taping of flexor region; immediate and 1.5 hours (P = 0.36) in taping of extensor region; immediate and 1 hour (P = 1.15), immediate and 1.5 hours (P = 0.16) in taping of flexor/extensor regions. All other comparisons were statistically significant at P > 0.05 for all 25 cases.

Data analysis using Repeated measurements analysis showed a significant difference between different times in females (P = 0.000). Further analysis using Bonferroni correction for Post Hoc tests revealed no significant differences between immediate and 0.5 hour (P = 1.00), 1 hour and 2 hours (P = 0.87) in taping of flexor region, 1 hour and 2 hours (P = 0.23) in taping of flexor/extensor regions in females. All other comparisons were statistically significant at P > 0.05 for all 28 cases.

**Figure 1.**
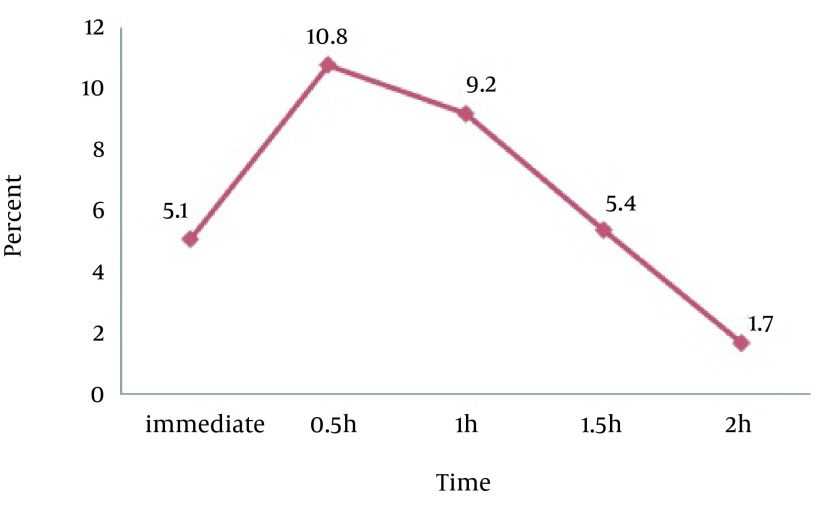
Percent Changes in Grip Strength After Kinesio Taping on Extensor Region in Males at Different Times

**Figure 2.**
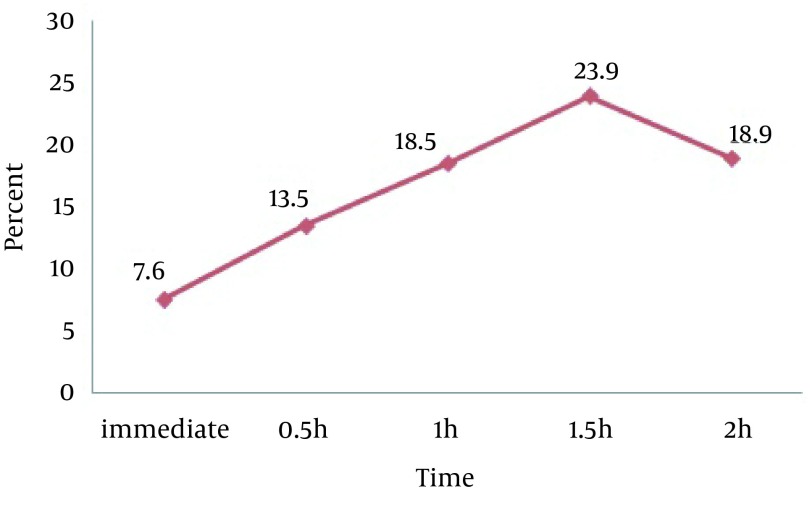
Percent Changes in Grip Strength After Kinesio Taping on Extensor Region in Females at Different Times

In conclusion, the greatest increase in grip strength occurred at 0.5 h of taping in males (P = 0.001) (Figure 1) and 1.5 hours in females (P = 0.000) (Figure 2).

Repeated measurements analysis demonstrated a significant difference between different regions after taping in males (P = 0.002). Further analysis using Bonferroni correction for Post Hoc tests revealed no significant differences between flexor and flexor/extensor regions in immediate (P = 1.01), 0.5 hour (P = 0.96), 1 hour (P = 0.72), and 1.5 hours (P = 1.00) after taping, flexor and extensor regions in 2 hours (P = 0.21), extensor and flexor/extensor regions in 2 hours (P = 0.17) after taping. All other comparisons were statistically significant at P > 0.05 for all 9 cases.

Repeated measurements analysis demonstrated a significant difference between different regions after taping in females (P = 0.000). Further analysis using Bonferroni correction for Post Hoc tests revealed no significant differences between flexor and flexor/extensor regions at 0.5 hour (P = 0.98), 1 hour (P = 1.00), 1.5 hours (P = 0.83), 2 hours (P = 0.76) after taping, All other comparisons were statistically significant at P > 0.05 for all 11 cases.

In general, among all three regions, taping of the extensor region of the forearm led to the highest improvement in grip strength in both men (up to 10.8%; P = 0.000) and women (up to 23.9%; P = 0.000).

## 5. Discussion

Our findings revealed signiﬁcant improvements in maximal grip strength immediately, 0.5, 1, 1.5, and 2 hours after Kinesio taping in flexor, extensor, and flexor/extensor regions of the forearm. In addition, there was a significant association between grip strength and taping region (flexor, extensor and flexor/extensor), i.e. in both genders, taping of the extensor region increased the maximal grip strength significantly compared to taping of the flexor and flexor/extensor regions. Furthermore, maximum grip strength improvement in men and women was observed after 0.5 and 1.5 hours of taping, respectively (Figures 1 and 2).

The results of the present study contradicted those obtained by Chang et al. (21) and Kuo et al. (22), but were in line with the findings of Lee et al. (23) and Donec et al. (24).

Chang et al. assessed immediate effect of Kinesio taping in flexor muscles of the wrist on the maximum grip strength. Twenty-one healthy male student athletes were evaluated in three different states as; with Kinesio taping, no taping, and with placebo taping. Y shape application from insertion to origin with light (15%-20%) stretch was used for Kinesio taping. They concluded that Kinesio taping had no effect on the maximum grip strength in healthy people (21). In a study on 19 healthy students (11 women and eight men), Kuo et al. applied inhibition and facilitation methods of Kinesio taping on dominant and non-dominant dorsal of forearm and hand, respectively. I and Y shape applications with 10% stretch were used in facilitation and inhibition methods, respectively. They measured grip strength before taping, immediately and 24 hours afterward (with the tape in situ). They reported that Kinesio tape did not affect grip strength (22). Apparently, the results of these two studies are in contrast with our findings. Regardless of the group of muscles taped and the direction of taping, it seems that Y shape application and inadequate stretch in the mentioned studies could lead to insufficient stimulation of afferent fibers and failure to enhance grip strength. According to Kase et al. (12) while 50%-75% stretch is warranted for muscular facilitation, muscular inhibition requires 15%-25% stretch.

Lee et al. studied grip strength in 20 healthy men and 20 healthy women in three groups of neutral head and neck alignment, head and neck turned to the non-dominant arm on the transverse plane, and flexor muscles of the dominant hand being Kinesio taped. The Kinesio tape was applied from origin to insertion with 15%-25% stretch. In both genders, grip strength was significantly higher in the Kinesio tape group than the other two groups (23). In a study to measure grip strength of 54 healthy, non-athlete male and female individuals, Donec et al. allocated subjects to three groups of Kinesio tape, placebo, and control. Y shape application on flexor and extensor muscles of fingers, from origin to insertion, with 15%-25% stretch was used in the Kinesio tape group. Grip strength values after 30 and 60 minutes showed that while Kinesio tape significantly increased grip strength at both times, no change was detected in placebo and control groups (24). These two studies confirm our findings regarding the effects of Kinesio tape on increasing grip strength.

Given insufficient information about the effects of Kinesio tape on proprioception, the tape seems to exert facilitating effects on mechanoreceptors (25). Previous studies suggested that through skin stretching, Kinesio tapes increase mechanoreceptor stimulation and sensory feedback of the taped region, facilitate muscle contraction, and ultimately promote muscle strength (26). Furthermore, facilitating effects of Kinesio tapes on mechanoreceptors reach their peak at joint active range (mid-range) where mechanoreceptors of ligaments are relatively inactive (25).

Based on the results of the present study, taping extensor region, rather than flexor or extensor/flexor regions of the forearm had the greatest effects on grip strength. Previous studies showed that extensor muscles stabilize the grip, exert compressive force on metacarpophalangeal and wrist joints and balancing force on flexors, and cause stronger contraction of flexor muscles by maintaining the length-tension relationship (27). On the other hand, extensor and flexor muscles have postural (tonic) and phasic nature, respectively. Due to lower stimulation threshold of type I muscle fibers (slow twitch) in tonic muscles compared to phasic muscles (28), taping stimulating low threshold receptors has a greater effect on extensor muscles. Moreover, the extensor side of the forearm is more hairy than its flexor side. More hair follicles under the tape can enhance muscle stimulation (29). Therefore, greater grip strength following taping of extensor muscles of the forearm can be attributed to tonic nature of these muscles and presence of more hair follicles in the area.

As mentioned before, the greatest effect of taping appeared after 0.5 hour in men and 1.5 hours in women. Since women generally enjoy better thermoregulation than men, their skin temperature is lower than men in low ambient temperature. Besides, their skin temperature increases more slowly than men. However, the ultimate temperature rise is greater in women than men (30). This process can justify delayed effect of taping in women. Although the tape acts as a protective layer preventing heat transfer and increases skin temperature, gradual temperature change in women (caused by thicker subcutaneous fat layer) slows the increase in strength.

There were some weaknesses in this study including small sample size, sampling method, and studying only normal subjects. Strength points of this study included determining the best region of forearm taping to reach the maximum increase in grip strength. Moreover, this study identified peak time effect of Kinesio taping in both genders. Therefore, athletes in both genders can obtain a better benefit as a result.

The results of the present study showed that Kinesio taping could effectively increase grip strength in healthy people. The best region for taping was extensor region of the forearm. Moreover, despite slower increase of grip strength in women, final levels were much higher in women than men.
